# Development and Validation of the Parent-Initiated Motivational Climate in Individual Sport Competition Questionnaire

**DOI:** 10.3389/fpsyg.2019.00128

**Published:** 2019-02-05

**Authors:** Chris G. Harwood, Emine Caglar, Sam N. Thrower, Jonathan M. J. Smith

**Affiliations:** ^1^School of Sport, Exercise and Health Sciences, Loughborough University, Loughborough, United Kingdom; ^2^Faculty of Sport Sciences, Hacettepe University, Ankara, Turkey; ^3^Department of Life Sciences, University of Roehampton, London, United Kingdom; ^4^Adaptivemind Consultancy, London, United Kingdom

**Keywords:** sport parents, motivational climate, achievement goals, individual sports, competition

## Abstract

This paper presents a series of studies that progresses the development and validation of the Parent-Initiated Motivational Climate in Individual Sport Competition Questionnaire (MCISCQ-Parent). Study 1 examined the face and content validity of an initial pool of 26 items based on the principles of achievement goal theory and prior research. In Study 2, data from an adolescent sample of individual sport athletes was subjected to an exploratory factor analysis (EFA) of items pertaining to the perceived task and ego involving characteristics of fathers and mothers in the competition setting. Study 3 tested the factor structure of the MCISCQ-Parent through confirmatory factor analysis (CFA) in a further youth athlete sample. Following appropriate CFA-related modifications, good goodness of fit indices emerged for the father- (three factor-model) and mother-related (two factor-model) dimensions of motivational climate. In Study 4, a further CFA was conducted and provided additional evidence for the revised factor structure of the MCISCQ-Parent, convergent and discriminant validity, and internal consistency. Finally, Study 5 provided support for the concurrent validity of the MCISCQ-Parent by demonstrating significant relationships between MCISCQ-Parent subscales and task and ego orientation, athlete engagement, and perceived social support. In sum, we present the MCISCQ-Parent as a measure with promising psychometric properties, and specifically to those applied researchers interested in assessing the quality of motivation-related parental involvement perceived by young athletes in the competition setting.

## Introduction

Over the last decade there has been growing academic interest in parental involvement in youth sport settings (see Knight et al., 2017 for a review). Much of this research has focused on the behaviors parents’ display and the way they interact with their child before, during, and after youth sport competitions (see Holt et al., 2008; Dorsch et al., 2015; Tamminen et al., 2017). In particular, these studies have provided some evidence to suggest that parents engage in more behaviors during individual sports than team sports (see Dorsch et al., 2015) and illustrate how behavior during competition is influenced by intrapersonal (e.g., goals, empathy, knowledge, and experience) and situational or contextual factors (e.g., stage of the game, the score, and importance of the competition). It is through these on-going interactions, before, during, and after competition that parents communicate their views about the value of winning and losing, expectations regarding success and their perceptions of their child’s competence. One theoretical lens that has been used to study the influence that parents (and other social agents) have on children’s perceptions of their own ability and motivation within youth sport competitions is achievement goal theory (e.g., Dweck, 1986; Nicholls, 1989; Ames, 1992).

Nicholls’ (1989) achievement goal theory is one of the most widely used theoretical frameworks within the sport psychology literature and addresses the intrapersonal and situational factors which influence individuals’ cognitive perceptions of success and failure, their attributions, affective responses, and subsequent behaviors (i.e., task choice, effort, persistence) (Roberts, 2001; Smith et al., 2008). As such, individuals’ achievement goals within specific situations (i.e., goal involvement) are determined by an interaction between their goal orientations and the motivational climate created by key social agents (e.g., parents, coaches, peers) (Dweck and Leggett, 1988; Harwood et al., 2008).

At an intrapersonal level, Nicholls’ dichotomous model of achievement goals proposes that internal perceptions of ability (or competence) are crucial in achievement tasks and that individuals develop a dispositional proneness to conceive ability as task-orientated or ego-orientated (see Nicholls, 1989; Roberts, 2001). There is considerable evidence to suggest that high levels of task-orientation are associated with positive cognitive, affective, and behavioral outcomes, while high levels of ego-orientation are associated with neutral or less optimal outcomes particularly when perceptions of competence are low or not accompanied by task-based goals (see Biddle et al., 2003; Harwood et al., 2008).

At a situational level, Ames (1992) proposed two types of motivational climate that could concurrently influence an individual’s achievement goal state (i.e., task or ego involvement) in an achievement context (e.g., youth sport competition). A mastery/task-involving climate is created when social agents are perceived to place emphasis on self-referenced improvement, effort, and cooperative learning, while a performance/ego involving climate is created when there is a perceived focus on outcomes, the emphasis is placed on outperforming others, preferential treatment is seen to be given to high-level performers, and mistakes are punished (Seifriz et al., 1992). As such, motivational climates are established by a pattern of normative influences, evaluative standards, rewards and sanctions, interpersonal interactions, and values communicated by social agents (e.g., coaches, peers, parents) within achievement contexts (Smith et al., 2008).

There is currently a large body of research that has examined the impact of coach and teacher initiated motivational climates using Nicholls’ model, however, parent initiated motivational climates have received far less academic attention within the literature. For example, in a recent systematic review of 104 studies between 1990 and 2014, Harwood et al. (2015) found that less than 3% of studies measured perceptions of the parent initiated motivational climate. Despite this, there is currently initial evidence to suggest that parent initiated mastery/task-involving climates are positively associated with young athletes’ task orientated goals (White et al., 1998), mastery approach (and avoidance) goal orientations (Morris and Kavussanu, 2008), autonomous regulation (i.e., intrinsic motivation), higher self-esteem, lower levels of anxiety (O’Rourke et al., 2014), dispositional flow (Caglar et al., 2017), and sport enjoyment (Atkins et al., 2015). In contrast, young athletes’ perceptions of a parents’ performance/ego-involving climate have been linked to ego-orientated goals (White et al., 1998), extrinsic motivation, higher levels of anxiety (O’Rourke et al., 2014), and perfectionistic cognitions (Appleton et al., 2011). Taken together, these studies support the notion that parents play a crucial role in young athletes’ motivation and experiences in youth sport and may even have a more significant role than coaches from an achievement goal perspective (O’Rourke et al., 2014).

To assess parent-initiated motivational climate in sport, almost all of the aforementioned studies have used the Parent Initiated Motivational Climate Questionaire-2 (PIMCQ-2; White, 1998). Originally adapted from the Learning and Performance Orientations in Physical Education Classes Questionnaire (LAPOPECQ), the PIMCQ-2 is an 18-item inventory that assesses athletes’ perceptions of the mastery (i.e., learning/enjoyment) and performance-orientated climates (i.e., worry-conductive and success-without-effort) created by their parents using a three-factor structure. However, despite its widespread use, the PIMCQ-2 has not undergone rigorous psychometric testing [e.g., confirmatory factor analysis (CFA)] and some items appear to refer to the cognitive responses of participants (e.g., ‘I feel that my mother/father makes me worried about failing in sport’) and emotions (e.g., ‘I feel that my mother/father is most satisfied when I learn something new in sport’) rather than the actual parental behaviors and cues associated with mastery/task or performance/ego involving climates. Such concerns have led scholars to note the caveat that the PIMCQ-2 may tap into the correlates associated with motivational climates rather than the actual motivational climate itself (Duda and Whitehead, 1998).

Beyond psychometric properties, the PIMCQ-2 items cover various social dimensions of motivational climate in a broad and generic manner. For instance, items (e.g., ‘I feel that my mother/father encourages me to enjoy learning new sport skills) do not locate respondents within specific context (i.e., training vs. competition), sport (e.g., tennis, swimming) or even sport type (i.e., individual vs. team sport). As a result, the specific contextual circumstances in which sport achievement tasks are performed by participants have been rather overlooked by the literature that has centered on assessments of perceived motivational climate (see Harwood et al., 2008 for a review). This is problematic as recent qualitative research has highlighted how the achievement-related behaviors and actions of parents and their influence upon their child-athlete (e.g., Keegan et al., 2010; Knight et al., 2016) may be sensitive to context and situation.

The scientific opportunity here for applied researchers interested the role of parental influence upon youth sport motivation is to develop more fine-grained, theoretically driven measures of parental involvement. Such measures would ideally capture the perceived parent-initiated motivational climate within specific youth sport contexts where parents are in attendance (i.e., competition vs. training) and with additional consideration to specific sport types (i.e., individual or team sports). Indeed, the development of a parental climate measure for specific sport types (i.e., individual sport) and contexts (i.e., competition) would enable researchers to examine the motivational processes and psychosocial responses of young performers in a more situationally aligned manner (Van de Pol et al., 2012). In addition to this, it would also enable applied researchers and practitioners to more accurately evaluate sport parent interventions designed to optimize achievement motivation-related behavior and parent–child interactions within youth sport competitions (see Thrower et al., 2017).

Much of the motivational climate literature in sport has focused on team sports (see Harwood et al., 2015), and this is fundamentally due to original scales being developed from team sport contexts and with items relevant to sampled performers being in a team situation. The motivational climate in a competition setting for an individual sport athlete (e.g., swimmer, tennis player, track and field athlete) remains largely understudied. However, such an understanding, through appropriate measurement, appears to be most needed within individual sports given that parents frequently interact with their child before, during, and after competition largely as the sole, responsible adult/caregiver on site in such sports. This is an important contextual point about individual sports and the reliance on parental support given the absence of individual coaches or the typical club/community representative coach in an organized team setting. Additionally, there is evidence to suggest that individual sport athletes report higher levels of ego orientation in competition than team-sport athletes (Van de Pol et al., 2012) and higher ego orientation in competition than training (i.e., Harwood, 2002; Van de Pol and Kavussanu, 2011). Such goal-related sensitivities in individual sport competition settings render the climate around these individuals worthy of more precise investigation.

The purpose of this research, therefore, was to develop a measure of athletes’ perceptions of the parent-initiated motivational climate in sport competition with explicit relevance to individual sports. To this end, we conducted five studies to progress the development of a valid and reliable instrument for use by applied researchers. The purpose of Study 1 was to develop appropriate items and examine their face and content validity. Study 2 aimed to determine the factor structure of the Parent-Initiated Motivational Climate in Individual Sport Competition Questionnaire (MCISCQ-Parent) through exploratory factor analysis (EFA). Studies 3 and 4 aimed to test the factor structure of the MCISCQ-Parent through CFA on separate, independent samples. The aim of the final study was to explore concurrent validity of the MCISCQ-Parent in relation to constructs and factors that are relevant to the achievement-related youth sport experience, namely achievement goal orientations, athlete engagement and social support.

## Study 1

Based on DeVellis’ (2012) recommendations for scale development, the aim of Study 1 was to generate a pool of items, which comprehensively captured the parent initiated motivational climate in individual sport competitions and to examine item content and face validity. A secondary aim of this study was to examine whether the developed items were applicable to junior individual sport athletes.

### Initial Item Generation

Following a standardized procedure for the creation of a new measure (see MSSYS, Smith et al., 2008; PeerMCYSQ; Ntoumanis and Vazou, 2005), a range of items were generated which reflected a parent-initiated task or ego involving motivational climate in a competition setting. Specifically, 18-items (10 task involving and 8 ego involving items) were generated from previous measures (e.g., PIMCQ-2; White, 1998) and relevant literature in individual sports (e.g., Harwood and Swain, 2001, 2002). Identical items were generated for the father and mother. The anchors “*Never occurs*” to “*Always occurs*” were used on a 7-point Likert scale to assess how often parents were perceived to conduct themselves in ways deemed representative of a task and ego involving climate.

### Methods

#### Participants

Institutional ethical approval, informed assent/consent and parental consent (if applicable) were obtained for all of the studies reported in this article. An expert panel was recruited to assess content and face validity of the generated items (Dunn et al., 1999). The expert panel comprised the six academics (i.e., two professors, four lecturers in Sport and Exercise Psychology) with a particular academic interest in both achievement motivation and motivational climates in sporting contexts. Overall, the expert panel (four males and two females) had between 5 and 28 (*M* = 14.16, *SD* = 10.18) years of experience working in academia. In addition to this, a usability panel was recruited to explore the applicability of the items to junior individual sport athletes (Haynes et al., 1995). Twelve athletes aged between 13 and 17 years (*M*_age_ = 15.08, *SD* = 1.37) from individual sports (i.e., tennis and swimming) agreed to participate in this panel. These athletes were recruited from professionally coached club programs and followed organized training and competition schedules.

#### Procedure

##### Expert panel

Following initial item generation, content and face validity of the items were examined by the expert panel. Experts were informed of the reason behind the development of the MCISCQ-Parent, the context and structure of the questionnaire and the instructions for examining the content validity of the items. Specifically, experts were asked to: (1) classify the items into one of three categories (i.e., ‘task involving climate,’ ‘ego involving climate’ or ‘neither’), (2) evaluate how well the item captured the targeted structure using a five-point Likert scale (1 = *not representative*; 5 = *highly representative*); and (3) assess the grammatical clarity of each item using a five point Likert scale (1 = *not grammatically sound*; 5 = *grammatically sound*). Experts worked independently of each other and were provided with the opportunity to make any suggestions that they felt might help to improve each item or suggest new items.

##### Usability panel

The appropriateness of each item was further explored via a usability panel to ensure that the items could be easily understood by the target audience, represented the intended construct, and were applicable to individual sports (DeVellis, 2012). The procedure for this panel consisted of three stages. Participants were: (1) provided with definitions of task (A) and ego (B) involving motivational climates; (2) asked to classify each item into one of three categories (i.e., A, B, neither) in order to ascertain the percentage agreement (Wang, 2001, Unpublished); and (3) asked to assess the items for clarity by underlining any words or phrases that they felt were unclear or confusing. Based on their responses, verbal probing techniques drawn from the cognitive interviewing method (Dietrich and Ehrlenspiel, 2010) were employed to further examine item comprehension (e.g., are there any statements that you find confusing or difficult to understand?) and applicability (e.g., are there any statements that you think do not relate to your environment or to athletes in similar situations to yourself?). Finally, participants were asked to complete the first page of the questionnaire and comment on the clarity of the instructions and the format of the questionnaire.

#### Analysis and Results

Retention of items was approved at this stage if: (a) there was 80% agreement (Eys et al., 2009) of the items onto the same construct; (b) the item was rated as being highly representative of the construct’s content (*M* > 4/5); and (c) items were found to be grammatically sound (*M* > 4/5). From the expert panel, all 18 items passed these criteria and were consequently retained. However, the expert panel suggested a number of grammatical changes to items together with possible incorporation of eight new items. This led to the development of an additional four task and four ego items, creating a total pool of 26 items (14 task involving and 12 ego involving items; see Supplementary Material for item pool). Following the usability panel, no items failed to meet the 70% agreement criterion level (Wang, 2001, Unpublished) assigned to the classification stage. Despite this, when concern was raised by the participants regarding difficulty in understanding certain words during the interview, these words were changed (e.g., ‘reinforces’ was changed to ‘reminds’).

## Study 2

The aim of the Study 2 was to test the initial factor structure of the mother and father scales (14 task involving items, 12 ego involving items) generated in Study 1 through an EFA (DeVellis, 2012). This data-driven approach was preferred over a CFA as a first step given the limited prior research specifically on parent-initiated climates and the emergence of a three-factor model forming past measures in this population (White et al., 1998).

### Methods

#### Participants

Purposeful sampling was used to identify British national governing bodies, clubs, or organizations from individual sports. Three organizations (e.g., swimming, tennis, athletics) responded to an initial email invitation to participate in the research and subsequently acted as a gatekeeper by inviting individual sport athletes to participate in the research. A total of 292 individual sport athletes agreed to take part in this phase of the study. Participants included 120 tennis players, 123 swimmers, and 49 track and field athletes (144 males and 148 females; *M*_age_ = 14.32; *SD* = 1.85; *M*_participaion_ = 7.33; *SD* = 2.82; *M*_compete_ = 5.46; *SD* = 2.39).

#### Measures and Procedure

The 26-item MCISCQ-Parent was administered to participants following training sessions. As noted in Study 1, each item was rated on a 7-point Likert scale ranging from 1 (*Never occurs*) to 7 (*Always occurs*) to represent the pervasiveness of parental task and ego involving behaviors and characteristics perceived by athletes in the competition setting. Participants were informed verbally, and within the information sheet, that involvement in the study was voluntary and results would be strictly confidential.

#### Data Analysis

Prior to main analysis, Bartlett’s test of sphericity and Kaiser-Meyer-Olkin (KMO) sampling adequacy statistic were conducted to determine the suitability of the data for EFA. Following this, principal component analysis followed by varimax rotation was conducted for each of the two dimensions (i.e., mother and father) in order to identify the factors and refine the number of items of the MCISCQ-Parent (Field, 2011). Factors were retained with eigenvalues greater than 1.0 (Meyers et al., 2006) and factor loadings of 0.45 (Hu and Bentler, 1999).

### Results

Bartlett’s test of sphericity was significant for the father (χ^2^ = 4167.57, *p* < 0.001) and mother dimensions (χ^2^ = 4103.04, *p* < 0.001) indicating that the correlation matrix was appropriate for factor analysis. The KMO yielded a value of 0.90 for father dimension and 0.92 for mother dimension, which exceeded the criterion of 0.60 (Tabachnick and Fidell, 2013).

#### Father Dimension

Initial examination of the father dimension revealed seven items which did not meet the criterion for retention, leaving 19 items to be further analyzed by principal component analysis. After examining the rotated correlation matrix, three items initially cross-loaded on two factors and were subsequently removed. When the analysis was repeated no further items cross-loaded. The items removed were split evenly between items proposed to depict an ego and task-involving climate. A four-factor solution again resulted with eigenvalues greater than one with the factors accounting for 65.4% of the variance (see Table 1). Factor 1 (termed: ‘ego-promoting values and behaviors’) incorporated six items all of which pertained to the athlete’s perception of the father’s values or behaviors being focused on being better than the opponent. Factor 2 (termed: ‘task-promoting behaviors’) consisted of four items which reflected the athlete’s perception of the father emphasizing the importance of reviewing their skills, learning from their experiences, and working hard while giving his/her best. These items aligned with the father behaviorally promoting the importance of self-referenced mastery. Factor 3 (termed: ‘negative reactions to poor effort and performance’) included items reflecting a negative response toward the athlete if they either did not try their best, made a mistake, or did not beat their opposition. Finally, Factor 4 (termed: ‘task promoting values’) comprised of three items referring to the athlete’s perception that their father sees mistakes as a part of learning, values the athlete doing the best they can, and showing positive affect from their actions.

**Table 1:** Factor pattern loadings from exploratory factor analysis of the father dimension of the MCISCQ-Parent.

Items	Factors				
		
	1	2	3	4	*M (SD)*
For me to beat an opponent is something that is important to my father	0.868				3.66 (2.00)
My father gives me the feeling that being better than my opponents is something that is important to him	0.806				3.23 (1.91)
Doing better than opponents or rivals is important to my father, and this is reflected in what he says to me	0.789				3.45 (1.93)
My father is concerned about whether or not I’m going to beat the opposition	0.744				3.24 (1.96)
My father compares my performance with the performance of other players/competitors	0.709				3.58 (2.03)
My father is proud of me if I show greater skills or strengths than my opposition	0.661				5.50 (1.70)
My father encourages me to review how I performed to help me learn from competition		0.852			4.47 (2.06)
My father is a big believer in helping me to understand my strengths in order to make progress		0.799			4.63 (1.85)
Before competition, my father reminds me of the importance of me trying my best		0.772			5.29 (1.96)
Before performing, my father gives me the feeling that succeeding is about working hard, learning and showing that I have made progress		0.637			4.84 (1.83)
My father pays no attention to me if I give up trying my best			0.799		2.65 (1.89)
My father is disappointed in me if I do not put in 100% effort			0.765		4.74 (2.08)
My father is annoyed if I make a mistake when performing			0.655		2.49 (1.67)
My father is happy with me if I have tried my best despite the result				0.770	6.08 (1.52)
My father is the kind of person who just wants me to perform to the best of my ability				0.761	6.11 (1.34)
My father views mistakes as part of learning				0.626	5.36 (1.62)


*Only loadings >0.45 are provided.*

#### Mother Dimension

Five items were removed from the analysis at the outset due to low factor loadings leaving 21 items. Following the varimax rotation three items were then discovered to cross load on two or more factors. These items were removed leaving a total of 18 items (see Table 2), consisting of three factors with eigenvalues greater than one and accounted for 60.5% of the variance. Factor 1 (termed: ‘ego promoting values and behaviors’) contained seven items with loadings between 0.84 and 0.62 and indicated the perceived values of the mother and the importance of the athlete being better than the opponent or beating the opponent. This factor also included items that described the behaviors of the mother that reflected an ego-involving climate. Factor 2 (i.e., ‘task promoting values and behaviors’) consisted of eight items loading between 0.75 and 0.64 reflecting the athlete’s impression that the mother values the athlete working hard, improving personal performance, and learning from either mistakes or competition. This factor also included items pertaining to maternal behavior before and after competition through reminding the athlete to try their best or through encouraging the athlete to review how they performed and what they learned from competition. Factor 3 (termed: ‘negative reactions to poor effort and performance’) related to the athlete perceiving that the mother was annoyed if the athlete lost, made a mistake or did not put in a 100% effort. This factor contained three items with loadings between 0.73 and 0.46.

**Table 2:** Factor pattern loadings from exploratory factor analysis of the mother dimension of the MCISCQ-Parent.

Items	Factors			
		
	1	2	3	*M (SD)*
To my mother, success is about being better than your opponent to other competitors	0.839			3.37 (1.91)
Doing better than opponents or rivals is important to my mother, and this is reflected in what she says to me	0.816			3.30 (1.91)
My mother is concerned about whether or not I’m going to beat the opposition	0.808			3.33 (1.89)
My mother compares my performance with the performance of other players/competitors	0.803			3.45 (1.90)
My mother gives me the feeling that being better than my opponents is important to her	0.801			3.17 (1.85)
For me to beat an opponent is something that is important to my mother	0.795			3.68 (1.86)
My mother is proud of me if I show greater skills or strengths than my opposition	0.617			5.21 (1.74)
Prior to performing, my mother gives me the feeling that succeeding is about working hard, learning and showing that I have made progress		0.745		4.89 (1.87)
My mother likes it when I improve my personal performance		0.722		6.04 (1.28)
My mother is a big believer in helping me to understand my strengths in order to make progress		0.713		4.60 (1.91)
My mother encourages me to review how I performed to help me learn from competition		0.706		4.61 (1.98)
My mother views mistakes as a part of learning		0.667		5.41 (1.58)
Before competition, my mother reminds me of the importance of me trying my best		0.659		5.62 (1.73)
My mother is the kind of person who just want me to perform to the best of my ability		0.656		6.26 (1.26)
My mother is keen to find out whether I played well or improved		0.638		5.92 (1.33)
My mother is annoyed if I make a mistake when performing			0.731	2.44 (1.62)
My mother rewards me only if I beat the opposition			0.671	2.32 (1.72)
My mother is disappointed in me if I don’t put in 100% effort			0.461	4.56 (2.13)


*Only loadings >0.45 are provided.*

## Study 3

The aim of Study 3 was to further the psychometric development and structure of the revised MCISCQ-Parent by testing the factorial validity of the measure. In this study, a number of CFAs were conducted using a modification analysis approach to determine the most sound and reliable construction of each of the dimensions.

### Methods

#### Participants and Sampling

Following the sampling procedures outlined in Study 2, a total of 398 male (*N* = 195) and female (*N* = 203) individual sport athletes between the ages of 13 and 23 years (*M* = 18.60; *SD* = 2.52) agreed to participate in this study. Athletes had between 1 and 18 years of sport experiences (*M*_participation_ = 8.09; *SD* = 4.06 years; *M*_compete_ = 6.37; *SD* = 3.53 years) and participated in a range of sports including tennis (*N* = 152), triathlon (*N* = 51), athletics (*N* = 47), swimming (*N* = 44), golf (*N* = 41), taekwondo (*N* = 38), ballet (*N* = 15), badminton (*N* = 7), and fencing (*N* = 3).

#### Measure and Procedure

All participants were administered the revised 34 item MCISCQ-Parent (18 items – mother dimension; 16 items – father dimension) following training sessions.

#### Data Analysis

Responses to the father and mother dimensions of the MCISCQ-Parent were analyzed independently. A CFA on each of the dimensions was conducted using AMOS Version 7.0 (Arbuckle, 2006), while Cronbach coefficient alpha (Cronbach, 1951) was used to examine internal reliability. Comparative Fit Index (CFI), Incremental Fit Index (IFI), Standardized Root Mean Square Residual (SRMR), and chi-square were utilized in the CFA. CFI and IFI values approaching 0.95 are considered to reflect a highly satisfactory fit between the hypothesized model and the data, whereas values less than 0.05 indicate a good fit for the SRMR (Hu and Bentler, 1999). As the chi-square (χ^2^) tests of fit are very sensitive to sample size, for a good model fit, the ratio χ^2^/df should be as small as possible. A ratio between 2 and 3 is indicative of a “good” or “acceptable” data-model fit, respectively (Schermelleh-Engel et al., 2003).

For each dimension an assessment of the normality of the data was undertaken through examining Mardia’s coefficient. In each dimension, this inspection revealed multivariate non-normality in the data. Therefore, analyses were performed using a bootstrapping technique (see Efron and Tibshirani, 1993). Bootstrapping was used as it is beneficial under conditions of non-normality as the bootstrap-generated standard errors provide a more accurate indication of the parameter estimate stability (Byrne, 2001; Nevitt and Hancock, 2001). Specifically, 5,000 bootstrap samples with replacement based on the original sample were requested. All subsequent CFAs were conducted using maximum likelihood estimation coupled with bootstrapping procedures (Preacher and Hayes, 2008; Sebire et al., 2009).

### Results

For the father dimension, a four-factor model with 16 items identified from the EFA was tested and the subsequent fit indices were examined. The indices suggested that there were still improvements needed for there to be appropriate confidence in the model structure (see Table 3). On examination of the modification indices, two items were removed (items 3 and 14). This led to factor 3 being removed due to the limited number of items within the factor and a subsequent CFA undertaken (see Table 3). Following a further examination of the modification indices, item 5 was also removed as it cross loaded and another CFA was undertaken on the remaining 11 items. This final CFA (see Table 3) resulted in a good fit between the three-factor structure and the observed data.

**Table 3:** Fit indices for father and mother CFA models.

Model	CFI	IFI	SRMR	χ^2^	df	χ^2^/df
*Father dimension*						
M1, 16 items	0.896	0.897	0.097	418.45	98	4.26
M2, 12 items	0.946	0.946	0.054	180.85	51	3.54
M3, 11 items	0.968	0.968	0.047	107.76	41	2.62
*Mother dimension*						
M1, 18 items	0.869	0.870	0.086	524.75	132	3.97
M2, 10 items	0.970	0.970	0.036	77.96	34	2.29


*All chi-square values are significant at the *p* < 0.001 level. M, model; CFI, Comparative Fit Index; IFI, Incremental Fit Index; SRMR, Standardized Root Mean Square Residual.*

Factor loadings were adequate and ranged from 0.70 to 0.85 (Hair et al., 2014) (see Table 4). Internal consistencies were also satisfactory for all factors with none being lower than 0.81 (Nunnally and Bernstein, 1994). Finally, the correlation between the two task promoting factors (termed: ‘task promoting behaviors’ and ‘task promoting values’) was moderate to strong (0.53) whereas associations between ‘task promoting values’ and ‘task promoting behaviors’ with the single ‘ego promoting values and behaviors’ factor were very weak (-0.06) and moderate (0.35), respectively.

**Table 4:** Descriptive statistics, squared multiple correlation, factor correlations, internal consistency, and standardized factor loadings of the father dimension of the MCISCQ-Parent.

				Factor				
						
	Father dimension subscales and items	*M*	*SD*	I	II	III	*SE*	SMC
	*Ego promoting values and behaviors*							
6	My father is concerned about whether or not I’m going to beat the opposition	3.74	1.89	0.72			0.034	0.52
8	For me to beat an opponent is something that is important to my father	3.97	1.87	0.81			0.031	0.66
10	My father gives me the feeling that being better than my opponents is something that is important to him	3.43	1.80	0.84			0.023	0.70
13	Doing better than opponents is important to my father, and this is reflected in what he says to me	3.57	1.79	0.85			0.023	0.71
	*Task promoting behaviors*							
1	Before competition, my father reminds me of the importance of me trying my best	5.06	1.90		0.70		0.041	0.49
2	My father encourages me to review how I performed to help me learn from competition	4.34	1.84		0.78		0.026	0.62
4	Before performing, my father gives me the feeling that succeeding is about working hard, learning and showing that I have made progress	4.47	1.85		0.80		0.026	0.64
11	My father is a big believer in helping me to understand my strengths in order to make progress	4.64	1.75		0.79		0.029	0.62
	*Task promoting values*							
7	My father is happy with me if I have tried my best despite the result	5.65	1.60			0.74	0.046	0.54
9	My father views mistakes as part of learning	5.02	1.64			0.74	0.040	0.55
12	My father is the kind of person who just wants me to perform to the best of my ability	5.77	1.50			0.77	0.038	0.59

				**Factor**				
						
	**Factor correlations and internal consistency coefficients**	***M***	***SD***	**I**	**II**	**III**		

I.	Ego promoting values and behaviors	3.68	1.58	0.88				
II.	Task promoting behaviors	4.63	1.52	0.35	0.87			
III.	Task promoting values	5.48	1.33	-0.06	0.53	0.81		


*Numbers to the left of each item represent the item’s position in the father dimension of the MCISCQ-Parent. SE, standard error; SMC, squared multiple correlation. Cronbach’s alpha coefficients are on the principle diagonal of the factor correlation matrix. All factor loadings are statistically significant (*p* < 0.05).*

Following the EFA, the mother dimension consisted of three factors and 18 items. A CFA was undertaken and the fit indices also indicated a need to improve the model. An inspection of the modification indices showed items 3 and 10 from factor 1, items 9, 13, and 15 from factor 2, and item 16 from factor 3 were highly cross loaded and were subsequently removed. Following the removal of item 16, factor 3 in its entirety was removed (items 17 and 18), due to the lack of stability of factors with two items (Ullman, 2007). The subsequent two-factor structure and ten items were then reanalyzed via CFA and resulted in an improved goodness of fit (see Table 3).

As shown in Table 5, factor loadings ranged from 0.55 to 0.85, indicating adequate loadings (Hair et al., 2014). Internal consistencies were acceptable for both factors – Factor 1 termed ‘ego promoting values and behaviors’ (0.90); Factor 2 termed ‘task promoting values and behaviors’ (0.85). The correlation between the factors was small to medium in strength (*r* = 0.32). In summary, Study 3 generated a 21-item questionnaire with three subscales for father dimension (11 items) and two subscales for mother dimension (10 items).

**Table 5:** Descriptive statistics, squared multiple correlation, factor correlations, internal consistency, and standardized factor loadings of the mother dimension of the MCISCQ-Parent.

				Factor			
						
	Mother dimension subscales and items	*M*	*SD*	I	II	*SE*	SMC
	*Ego promoting values and behaviors*						
4	To my mother, success is about being better than your opponent or other competitors	3.51	1.73	0.71		0.041	0.50
6	My mother compares my performance with the performances of other players/competitors	3.60	1.83	0.69		0.035	0.47
7	My mother is concerned about whether or not I’m going to beat the opposition	3.59	1.86	0.79		0.028	0.63
8	For me to beat an opponent is something that is important to my mother	3.62	1.78	0.85		0.027	0.72
14	Doing better than opponents or rivals is important to my mother, and this is reflected in what she says to me	3.46	1.76	0.68		0.042	0.46
	*Task promoting values and behaviors*						
1	Before competition, my mother reminds me of the importance of me trying my best	5.28	1.74		0.60	0.050	0.36
2	My mother encourages me to review how I performed to help me learn from competition	4.06	1.92		0.78	0.030	0.61
5	Before performing, my mother gives me the feeling that succeeding is about working hard, learning and showing that I have made progress	4.51	1.76		0.63	0.048	0.40
11	My mother is a big believer in helping me to understand my strengths in order to make progress	4.53	1.79		0.75	0.038	0.56
12	My mother is keen to find out whether I played well or improved	5.62	1.54		0.55	0.049	0.30

				**Factor**			
						
	**Factor correlations and internal consistency coefficients**	***M***	***SD***	**I**	**II**		

I.	Ego promoting values and behaviors	3.57	1.44	0.90			
II.	Task promoting values and behaviors	4.68	1.35	0.32	0.85		


*Numbers to the left of each item represent the item’s position in the mother dimension of the MCISCQ-Parent. SE, standard error; SMC, squared multiple correlation. Cronbach’s alpha coefficients are on the principle diagonal of the factor correlation matrix. All factor loadings are statistically significant (*p* < 0.05).*

## Study 4

Given the results of Study 3, the purpose of Study 4 was to investigate the revised factor structure with a second CFA on a separate sample to ensure that the new model did not capitalize on the idiosyncrasies of a particular sample (Hoyle and Panter, 1995). A secondary aim of this study was to explore the convergent and discriminant validity of the MCISCQ-Parent using procedures proposed by Fornell and Larcker (1981).

### Methods

#### Participants and Sampling

Consistent with the sampling criteria used in Studies 2 and 3, a total of 251 athletes (142 males, 131 females) agreed to participate in the study from a variety of individual sports including tennis, athletics, swimming, badminton, boxing, and gymnastics. Participants were aged between 15 and 26 years (*M* = 20.1, *SD* = 2.20 years) and has between 1 and 18 years experience in their sport (*M*_participation_ = 9.57; *SD* = 3.52 years; *M*_compete_ = 7.60; *SD* = 3.25 years).

#### Measure and Procedure

Consistent with studies two and three, participants were administered the final 21-item of MCISCQ-Parent following training sessions.

#### Data Analysis

In line with the data analysis procedures used in Study 3 the two dimensions of the MCISCQ-Parent were analyzed independently. An assessment of normality was undertaken and due to the non-normality of the data the same bootstrapping technique was applied. The goodness-of-fit indices were utilized to evaluate the adequacy of the factorial structure of the measure including the CFI, IFI, SRMR, and chi-squared tests. A CFA on each of the dimensions was undertaken along with Cronbach’s coefficient alpha to examine the internal reliability (Cronbach, 1951). The first order factor model for each dimension was tested as identified by the CFAs undertaken in Study 3.

In addition, the average variance extracted (AVE) was examined using Fornell and Larcker’s (1981) guidelines to test the convergent validity of the MCISCQ-Parent. AVE was set at 0.5 or greater to achieve adequate convergent validity, while standardized factor loadings were set at 0.5 or higher, and ideally 0.7 or higher (Hair et al., 2014). Discriminant validity was also investigated through comparisons of AVE and squared correlations between pairs of latent variables (Fornell and Larcker, 1981). This comparison indicates the extent to which each construct is more closely related to its own measures than those of other constructs. Based on Fornell and Larcker’s (1981) suggestions, AVE should be greater than the squared correlations between latent factors to provide evidence of discriminant validity.

### Results

Results revealed a good goodness of fit across all dimensions and were consistent with those found in Study 3 with each hypothesized model demonstrating a good fit with the observed data (see Table 6). The levels for both dimensions were slightly lower for each of the fit indices but still satisfactory in relation to cut off values (Hu and Bentler, 1999).

**Table 6:** Fit indices for models of the MCISCQ-Parent.

Dimensions	CFI	IFI	SRMR	χ^2^	df	χ^2^/df
Father (11 items)	0.961	0.962	0.044	103.22	41	2.51
Mother (10 items)	0.959	0.959	0.049	87.66	34	2.57


*All chi-square values are significant at the *p* < 0.001 level. CFI, Comparative Fit Index; IFI, Incremental Fit Index; SRMR, Standardized Root Mean Square Residual.*

#### Convergent and Discriminant Validity

Table 7 highlights that the AVE of the MCISCQ-Parent subscales exceeded the recommended level of 0.5 and all composite scale reliabilities exceeded the desired cut off of 0.7, showing adequate convergence (Hair et al., 2014). In addition, all standardized factor loadings (see Table 8) were >0.5 ranging from 0.71 to 0.90 for the father dimension and from 0.68 to 0.88 for the mother dimension, indicating good convergent validity. Furthermore, all AVE values (see Table 7) were greater than the squared correlations (off-diagonal), supporting the discriminant validity of the MCISCQ-Parent.

**Table 7:** The composite reliability (in bold), AVE, and squared correlations between subscales (off-diagonal) of MCISCQ-Parent.

	AVE	Ego promoting values and behaviors	Task promoting behaviors	Task promoting values
*Mother dimension*				
Ego promoting values and behaviors	0.63	**0.89**		
Task promoting values and behaviors	0.56	0.15	**0.86**	
*Father dimension*				
Ego promoting values and behaviors	0.70	**0.91**		
Task promoting behaviors	0.59	0.31	**0.85**	
Task promoting values	0.61	0.02	0.34	**0.82**


*AVE, average variance extracted.*

**Table 8:** Descriptive statistics, standardized factor loadings, squared multiple correlation, factor correlations and internal consistency of the MCISCQ-Parent.

	MCISCQ-Father								MCISCQ-Mother					
			
			Factors								Factors			
											
Subscales and items	*M*	*SD*	I	II	III	*SE*	SMC	Subscales and items	*M*	*SD*	I	II	*SE*	SMC
*Ego promoting values and behaviors*								*Ego promoting values and behaviors*						
6	3.55	1.97	0.79			0.037	0.62	4	3.64	1.75	0.71		0.047	0.49
8	3.79	1.90	0.90			0.019	0.82	6	3.82	1.83	0.77		0.035	0.59
10	3.39	1.81	0.86			0.030	0.74	7	3.82	1.86	0.88		0.022	0.77
13	3.47	1.83	0.81			0.039	0.66	8	3.66	1.86	0.88		0.027	0.77
								14	3.50	1.79	0.71		0.047	0.50
*Task promoting behaviors*								*Task promoting values and behaviors*						
1	4.39	2.02		0.71		0.039	0.50	1	4.89	1.88		0.73	0.044	.53
2	3.84	1.87		0.78		0.032	0.60	2	4.09	1.86		0.83	0.029	0.68
4	4.14	1.85		0.83		0.025	0.69	5	4.46	1.73		0.71	0.049	0.50
11	4.22	1.88		0.76		0.035	0.57	11	4.43	1.84		0.79	0.035	0.63
*Task promoting values*								12	5.30	1.67		0.68	0.051	0.46
7	5.32	1.77			0.80	0.046	0.64							
9	4.67	1.82			0.72	0.048	0.51							
12	5.38	1.75			0.82	0.040	0.66							

**Factor correlations and internal consistency coefficients**														

	***M***	***SD***							***M***	***SD***				

I. Ego promoting values and behaviors	3.55	1.66	0.91					I. Ego promoting values and behaviors	3.69	1.52	0.89			
II. Task promoting behaviors	4.15	1.58	0.56	0.85				II. Task promoting values and behaviors	4.57	1.45	0.39	0.86		
III. Task promoting values	5.12	1.52	0.15	0.58	0.82									


*Numbers to the left of each item represent the item’s position in the father dimension of the MCISCQ-Parent. SE, standard error; SMC, squared multiple correlation. Cronbach’s alpha coefficients are on the principle diagonal of the factor correlation matrix. All factor loadings are statistically significant (*p* < 0.05).*

Internal consistencies for both dimensions exceeded Nunnally and Bernstein’s (1994) 0.7 cut off value for all factors with none being lower than 0.82 (see Table 8). The correlation between the two task promoting factors (i.e., ‘task promoting behaviors’ and ‘task promoting values’) was moderate to strong (0.58) for the father dimension. For ‘ego promoting values and behaviors’ there was a weak correlation with the ‘task promoting values’ factor (0.15) and a moderate to strong correlation with the ‘task promoting behaviors’ factor (0.56) which was greater than the correlation reported in Study 3. In the mother dimension, the correlation between the two factors was small to moderate in strength (0.39).

## Study 5

The aim of Study 5 was to test the concurrent validity of the MCISCQ-Parent through correlations with a range of dispositional and situational factors relevant to the achievement context of youth sport. To this end, the relationships between parent-initiated motivational climate, athlete achievement goal orientations (Cumming et al., 2008), perceived social support (Alfermann et al., 2005; Sheridan et al., 2014) and athlete engagement (Curran et al. (2015) were explored. These factors would inform the validity of the MCISCQ-Parent in terms of associations with athletes’ individual differences, quality of parental support, and cognitive-affective experiences of sport.

Relationships between perceived coach created motivational climate and dispositional goal orientation are well established in sport settings. Research has found a mastery/task involving climate to be positively associated with task goal orientation while perceptions of an ego-involving motivational climate are significantly related to ego goal orientation (Smith et al., 2009; Harwood et al., 2015).

As constructs that both represent perceptions of the context, previous research has shown mastery climates to be positively associated with perceived social support in sport (Alfermann et al., 2005; Stanger et al., 2018) and perceived teacher support in physical education settings (Cox and Williams, 2008). Support derived from key interpersonal relationships (e.g., coaches, parents, peers) in a sporting context has been identified as an important resource for athletes (Sheridan et al., 2014). Levels of esteem and emotional support from parents through encouragement of effort, praise and empathy with their child are particularly pertinent to explore in terms of differential relationships with parental task and ego involving climates.

Finally, the recent empirical focus on athlete engagement as a cognitive-affective experience comprised of athlete perceptions of confidence, vigor, dedication and enthusiasm for sport activity has extended to the potential role of motivational climate. Curran et al. (2015) noted how a mastery climate may facilitate the perceptions of control that are instrumental to athlete engagement. In support of this notion, the authors reported that all dimensions of engagement were positively associated with perceptions of a coach-created mastery climate, with only the cognitive aspects of engagement correlated with performance climate. Hence it was viewed as timely and relevant to explore engagement experiences through the lens of the climate created by parents around competition.

### Method

#### Participants and Sampling

A purposeful sample of 92 tennis players was recruited for the current study. Specifically, 59 males and 33 females between the ages of 14 and 23 years (*M*_age_ = 16.45, *SD* = 1.59 years) agreed to participate in this study. Athletes had between 4 and 17 years of tennis experiences (*M* = 10.40; *SD* = 2.58 years).

#### Measures and Procedure

All participants completed a multi-section inventory online consisting of the final version of 21-item MCISCQ-Parent (see Appendix for final version), the Achievement Goal Scale for Youth Sport (AGSYS; Cumming et al., 2008), the Athlete Engagement Questionnaire (AEQ; Lonsdale et al., 2007), and the Perceived Available Support in Sport Questionnaire (PASS-Q; Freeman et al., 2011).

#### Data Analysis

An assessment of normality was conducted and due to non-normally distributed data Spearman’s correlation coefficient was used to investigate relationships between MCISCQ-Parent and measures of task and ego orientation, athlete engagement, and perceived social support (Field, 2011).

### Results

Descriptive statistics from the sample are presented in Table 9. The ‘task promoting behaviors’ and ‘task promoting values’ subscales of the MCISCQ-Father and the ‘task promoting values and behaviors’ subscale of MCISCQ-Mother were significantly and positively related with athlete task orientation, perceived social support, and athlete engagement (*p* < 0.05, *p* < 0.01) with the exception of the ‘confidence’ and ‘vigor’ subscales for the MCISCQ-Father and ‘enthusiasm’ subscale for the MCISCQ-Mother. Both fathers and mothers’ ‘ego promoting values and behaviors’ were significantly and positively associated with athletes’ ego orientation (*p* < 0.01) (see Table 9), with no relationships emerging for athlete engagement. In terms of perceived social support, athletes’ perception of their father’s ego promoting values and behaviors was negatively correlated to their perceived levels of esteem support (Table 9). Overall, the relationships between these variables showed promising support for the concurrent validity of the MCISCQ-Parent.

**Table 9:** Correlations between MCISCQ-Mother/Father and measures of task and ego orientation, athlete engagement, and perceived social support.

	Mean	*SD*	α	Ego promoting values and behaviors	Task promoting behaviors	Task promoting values
				M/F	M/F	F
*AGSYS*						
Task orientation	4.35	0.47	0.72	-0.01/0.002	0.34^∗∗^/0.27^∗^	0.23^∗^
Ego orientation	3.39	0.89	0.87	0.36^∗∗^/0.38^∗∗^	-0.01/0.10	0.03
*AEQ*						
Confidence	4.20	0.57	0.78	-0.04/-0.08	0.30^∗∗^/0.17	0.12
Dedication	4.55	0.53	0.85	0.03/-0.09	0.30^∗∗^/0.30^∗∗^	0.24^∗^
Vigor	4.46	0.56	0.86	0.12/-0.006	0.26^∗^/0.16	0.22^∗^
Enthusiasm	4.56	0.51	0.81	-0.06/-0.16	0.19/0.30^∗∗^	0.30^∗∗^
Total athlete engagement	4.44	0.45	0.92	-0.01/-0.10	0.34^∗∗^/0.31^∗∗^	0.28^∗∗^
*PASS-Q*						
Emotional support	4.71	0.41	0.81	-0.09/-0.05	0.40^∗∗^/0.38^∗∗^	0.47^∗∗^
Esteem support	3.98	0.86	0.88	-0.13/-0.25^∗^	0.47^∗∗^/0.29^∗∗^	0.30^∗∗^
Informational support	3.35	1.10	0.88	0.08/-0.04	0.36^∗∗^/0.38^∗∗^	0.17
Tangible support	4.30	0.64	0.58	-0.01/0.02	0.40^∗∗^/0.42^∗∗^	0.33^∗∗^
Mean				2.93/2.85	4.70/4.33	5.11
*SD*				1.20/1.26	1.20/1.32	0.98


*AGSYS, Achievement Goal Scale for Youth Sport; AEQ, Athlete Engagement Questionnaire; PASS-Q, Perceived Available Support in Sport Questionnaire; α, Cronbach’s alpha; M, MCISCQ-Mother; F, MCISCQ-Father, ^∗^*p* < 0.05, ^∗∗^*p* < 0.01.*

## Discussion

The aim of this series of studies was to address limitations in the assessment of parent-initiated motivational climate by developing and validating a measure of specific pertinence to parents of individual sport athletes in the context of competition. A series of five studies generated a 21-item questionnaire focused on the motivational climate created by mothers and fathers separately within competition contexts. The mother dimension consisted of two factors (i.e., ‘task promoting values and behaviors’ and ‘ego promoting values and behaviors’) with 10 items, while the father dimension consists of 3 factors (i.e., ‘ego promoting values and behaviors’, ‘task promoting behaviors’ and ‘task promoting values’) with 11 items. The MCISCQ-Parent has been validated for use with adolescent individual sport athletes between 13 and 23 years of age with mean readability age of Grade 7 (*M* = 7.46; *SD* = 2.04) (Flesch, 1949). As such, the MCISCQ-Parent can be used for both longitudinal or intervention research with athletes during adolescence (Grade 7 = 12–13 years old) and is brief enough to be used within a battery of measures administered to individual sport athletes.

Following item generation (Study 1), Study 2 used EFA to obtain a statistically sound structure for each of the dimensions. EFA results yielded four factors with 16 items for the father dimension and three factors with 18 items for the mother dimension. The emergence of different items and factors within the EFA supports the notion that mothers and fathers contribute to the child’s motivation in different ways (White, 2007). Within the context of sport, the dominant sport fathering role has been defined in terms of simply being involved (e.g., sharing the sport experience) compared to mothering roles which outline more intensive support of the sport experience (e.g., being responsible for child development and providing support; Trussel and Shaw, 2012).

In Study 3, a CFA was used to generate a revised model with a two-factor structure for the mother dimension and a three-factor structure for the father dimension. A second CFA confirmed these factor structures of the MCISCQ-Parent in an independent sample (Study 4). In contrast to previous measures of parent-initiated motivational climate (i.e., PIMCSQ-2; White, 1998) the final mother dimension included a two-factor structure while the father dimension included one ego involving factor and two task involving climate factors. The factor loadings of the mother and father dimensions of the final version MCISCQ-Parent ranged from 0.71 to 0.90 for the father dimension and 0.68 to 0.88 for the mother dimension (i.e., ranging from good to excellent (Tabachnick and Fidell, 2013). In addition to this, internal consistency coefficients of both dimensions of the MCISCQ-Parent were above 0.80 for all factors in both Studies 3 and 4. Kline (1993) suggests that although the generally accepted value of 0.80 represents acceptable reliability, for tests of psychological constructs a cut-off point of 0.70 is more suitable. Both of the mother and father dimensions of the MCISCQ-Parent had sufficient goodness of fit indices. Upon closer examination of the factors, findings suggest that fathers are perceived by their children to hold values associated with effort, development and learning within competition (i.e., task promoting values) but are less likely to engage in explicit task-involving behaviors. These findings support studies which have highlighted how mothers see themselves as giving more support and being more actively involved in athletes’ sport activities than fathers do (Wuerth et al., 2004). This role-related behavior may go some way to explaining why an inconsistency, or a lack of congruence, occurs between perceptions of fathers’ actual task promoting behaviors in competition set against the developmental values they are perceived to communicate in such a setting^1^.

Study 5 also found support for the concurrent validity of the MCISCQ-Parent by demonstrating significant correlations between MCISCQ-Parent subscales and the measures of task and ego orientation (Cumming et al., 2008), athlete engagement (Lonsdale et al., 2007), and perceived social support (Freeman et al., 2011). Specifically, findings demonstrated a positive correlation between perceived parent-initiated task involving climates and task orientation and no notable association between perceptions of an ego involving climate and task orientation. Links were also substantiated between parent-initiated task involving climates, overall athlete engagement, and perceived social support. In contrast, moderate positive associations between perceived parental ego involving climates and ego orientation emerged in addition to a negative relationship between perceptions of a paternal ego involving climate and available esteem support. The nature of these relationships provide further support for existing studies, which have examined the relationship between mastery climates and task orientation (see Harwood et al., 2015), athlete engagement (e.g., Curran et al., 2015) and perceived social support (e.g., Alfermann et al., 2005; Stanger et al., 2018).

Use of the MCISCQ-Parent should be considered in light of several limitations. Firstly, consistent with existing measures of parent-initiated motivational climate, the development and validation of the instrument was reliant on solely self-report data. Although the MCISCQ-Parent provides insights into athletes’ perceptions of the parent-initiated climate, future research should consider incorporating multiple methods (e.g., self-report and observation) to address the limitations of one method of assessment. Secondly, the data collected in this paper was cross-sectional and correlational in nature. Although this approach was appropriate for developing and validating the measure and examining pertinent relationships, future research should adopt longitudinal designs, which are more effectively able to examine how parental climates in the competition context may change over time (e.g., during a season; between transitions in stages of athlete development). Field-based intervention research with experimental and control groups of parents in relatively matched community settings would also further test the value and utility of this measure. Thirdly, the MCISCQ-Parent has been validated with athletes between the ages of 13 and 23 years of age, as a result we encourage future researchers to adapt the instrument and explore the reliability and validity of the measure for use with younger athletes (e.g., 10–12 years). Such developments represent a logical avenue for future research given that there is growing evidence to suggest that young athletes are able to cognitively differentiate between ability and effort on physical tasks from as young as 9 years of age (Smith et al., 2008). Considerate of attention spans for younger children, it may be useful for researchers to draw out and test a smaller cluster of task and ego involving items from the scale that have a lower reading age (e.g., My father views mistakes as part of learning). As validation is an on-going process, future studies are also encouraged to examine other salient forms of validity (e.g., discriminant validity through examining relationships with theoretically unrelated variables) as well as test-retest reliability. Another avenue for future research would be the examination of the factor structure of the MCISCQ-Parent through investigations of its cross-cultural invariance or measurement invariance across groups (e.g., gender, competitive level).

As a final point, the current paper presents a measure that is designed to capture the perceived parent-initiated motivational climate within specific youth sport contexts (i.e., competition) and aligned to specific sport types (i.e., individual sports). The measure has not been validated for use with team sports participants, and while we would encourage scholars to consider the applicability of the scale for such populations, it would be vital to assess the content validity and phrasing of each item for its meaningfulness in a team setting. This may assist in the development and testing of an appropriately adjusted scale for assessing the parent-initiated climate in team sports. Moving forward, we also believe that future research is needed to develop coach and peer- initiated motivational climate measures that are relevant and sensitive to similar contexts (e.g., individual sport situations). Such measurement advances are crucial to gain a more in-depth and finer grained understanding of the motivational climate that key social agents create in specific youth sport settings. This research would also help practitioners to more accurately evaluate sport parent interventions designed to optimize achievement motivation-related behavior and parent–child interactions within youth sport (see Thrower et al., 2017).

## Conclusion

In conclusion, the five progressive studies presented in this paper report the development and validation of the MCISCQ-Parent. The assessment of adolescents’ perceptions of the motivational climate created by mothers and fathers around individual sport competition is an important process and we hope that researchers find this tool valuable for progressing motivation-related youth sport research.

## Ethics Statement

This study was carried out in accordance with the recommendations of Loughborough University ethics committee with written informed consent from all subjects. All subjects gave written informed consent in accordance with the Declaration of Helsinki. The protocol was approved by the Loughborough University Ethics committee.

## Author Contributions

CH was responsible for the conceptualization and management of research program. JS, CH, and EC were involved with data collection and analysis. EC, ST, and CH were responsible for manuscript preparation.

## Conflict of Interest Statement

The authors declare that the research was conducted in the absence of any commercial or financial relationships that could be construed as a potential conflict of interest.

## Footnotes

1 As part of the peer review process, CFA’s were conducted on a two-factor (task and ego) solution for fathers in Studies 2 and 5 but the goodness of fit indices were poor in contrast to the positive results for the three-factor structure.
