# Supplementary material for: Development and Validation of the Parent-Initiated Motivational Climate in Individual Sport Competition Questionnaire
Source: Front Psychol. 2019 Feb 5;10:128. doi: 10.3389/fpsyg.2019.00128 (PMC6370734; doi:10.3389/fpsyg.2019.00128)
Supplement: Supplementary file 1 [file Table_1.DOCX]

**MCISCQ-Parent**

Taking part in competition can be quite a varied experience for all sports performers. Different people (e.g. mother, father, coach, other performers) can be involved in shaping your experiences as an individual competitor. We would like you to answer as accurately as possible how the statements below relate to your father and mother in terms of your sport competitions.

**Directions**: The questionnaire is divided into two sections – one related to your father, and one related to your mother. We would like to know the degree to which you generally believe that these statements about your parents occur, from ‘never occurs’ to ‘always occurs’, in your typical competition environment. There are **no right or wrong answers** and they will not be shared with anyone else so please answer as **honestly** as possible.

**This section is about your father/stepfather or male guardian. Please fill in this section if it applies to you.**

|  | | **Never occurs** | **Sometimes occurs** | | | | | **Always occurs** |
| --- | --- | --- | --- | --- | --- | --- | --- | --- |
| 1. | Before competition, my father reminds me of the importance of me trying my best. | 1 | 2 | 3 | 4 | 5 | 6 | 7 |
| 2. | My father encourages me to review how I performed to help me learn from competition. | 1 | 2 | 3 | 4 | 5 | 6 | 7 |
| 3. | Before performing, my father gives me the feeling that succeeding is about working hard, learning and showing that I have made progress. | 1 | 2 | 3 | 4 | 5 | 6 | 7 |
| 4. | My father is concerned about whether or not I'm going to beat the opposition. | 1 | 2 | 3 | 4 | 5 | 6 | 7 |
| 5. | My father is happy with me if I have tried my best despite the result. | 1 | 2 | 3 | 4 | 5 | 6 | 7 |
| 6. | For me to beat an opponent is something that is important to my father. | 1 | 2 | 3 | 4 | 5 | 6 | 7 |
| 7. | My father views mistakes as part of learning. | 1 | 2 | 3 | 4 | 5 | 6 | 7 |
| 8. | My father gives me the feeling that being better than my opponents is something that is important to him. | 1 | 2 | 3 | 4 | 5 | 6 | 7 |
| 9. | My father is a big believer in helping me to understand my strengths in order to make progress. | 1 | 2 | 3 | 4 | 5 | 6 | 7 |
| 10. | My father is the kind of person who just wants me to perform to the best of my ability. | 1 | 2 | 3 | 4 | 5 | 6 | 7 |
| 11. | Doing better than opponents is important to my father, and this is reflected in what he says to me. | 1 | 2 | 3 | 4 | 5 | 6 | 7 |

**This section is about your mother/stepmother or female guardian. Please fill in this section if it applies to you.**

|  | | **Never occurs** | **Sometimes occurs** | | | | | **Always occurs** |
| --- | --- | --- | --- | --- | --- | --- | --- | --- |
| 1. | Before competition, my mother reminds me of the importance of me trying my best. | 1 | 2 | 3 | 4 | 5 | 6 | 7 |
| 2. | My mother encourages me to review how I performed to help me learn from competition. | 1 | 2 | 3 | 4 | 5 | 6 | 7 |
| 3. | To my mother, success is about being better than your opponent or other competitors. | 1 | 2 | 3 | 4 | 5 | 6 | 7 |
| 4. | Before performing, my mother gives me the feeling that succeeding is about working hard, learning and showing that I have made progress. | 1 | 2 | 3 | 4 | 5 | 6 | 7 |
| 5. | My mother compares my performance with the performances of other players/competitors. | 1 | 2 | 3 | 4 | 5 | 6 | 7 |
| 6. | My mother is concerned about whether or not I'm going to beat the opposition. | 1 | 2 | 3 | 4 | 5 | 6 | 7 |
| 7. | For me to beat an opponent is something that is important to my mother | 1 | 2 | 3 | 4 | 5 | 6 | 7 |
| 8. | My mother is a big believer in helping me to understand my strengths in order to make progress. | 1 | 2 | 3 | 4 | 5 | 6 | 7 |
| 9. | My mother is keen to find out whether I played well or improved. | 1 | 2 | 3 | 4 | 5 | 6 | 7 |
| 10. | Doing better than opponents or rivals is important to my mother, and this is reflected in what she says to me. | 1 | 2 | 3 | 4 | 5 | 6 | 7 |

**Scoring Key:** Father dimension:

Ego Promoting Values and Behaviours: 4, 6, 8, 11

Task Promoting Behaviours: 1, 2, 3, 9

Task Promoting Values: 5, 7, 10

Mother dimension:

Ego Promoting Values and Behaviours:3, 5, 6, 7, 10

Task Promoting Values and Behaviours: 1, 2, 4, 8, 9
