# Supplementary material for: Development and Validation of the Parent-Initiated Motivational Climate in Individual Sport Competition Questionnaire
Source: Front Psychol. 2019 Feb 5;10:128. doi: 10.3389/fpsyg.2019.00128 (PMC6370734; doi:10.3389/fpsyg.2019.00128)
Supplement: Supplementary file 2 [file Table_2.DOCX]

**SUPPLEMENTARY MATERIAL**

FINAL POOL ITEMS OF MCISCQ-Parent for Study 2

1. Before competition, my father (mother*) reminds me of the importance of me trying my best. (T)
2. My father encourages me to review how I performed to help me learn from competition. (T)
3. My father praises me if I competed to the best of my ability. (T)
4. My father is proud of me if I show greater skills or strengths than my opposition. (E)
5. To my father, success is about being better than your opponent or other competitors. (E)
6. Before performing, my father gives me the feeling that succeeding is about working hard, learning and showing that I have made progress. (T)
7. My father compares my performance with the performances of other players/competitors. (E)
8. My father is concerned about whether or not I'm going to beat the opposition. (E)
9. My father is happy with me if I have tried my best despite the result. (T)
10. For me to beat an opponent is something that is important to my father. (E)
11. My father views mistakes as part of learning. (T)
12. My father encourages me if I give 100% effort. (T)
13. My father gives me the feeling that being better than my opponents is something that is important to him (her*) (E)
14. My father is a big believer in helping me to understand my strengths in order to make progress. (T)
15. My father is keen to find out whether I played well or improved. (T)
16. My father asks me about what position I came, or whether I won or lost. (E)
17. My father really values improvements that I make in my personal performance. (T)
18. My father is the kind of person who just wants me to perform to the best of my ability. (T)
19. Doing better than opponents or rivals is important to my father, and this is reflected in what he (she*) says to me. (E)
20. My father likes it when I improve my personal performance. (T)
21. My father ‘moans’ at me if I lose. (E)
22. When I lose my father is disappointed in me. (E)
23. My father is disappointed in me if I do not put in 100% effort. (T)
24. My father rewards me only if I beat the opposition. (E)
25. My father is annoyed if I make a mistake when performing. (T)
26. My father pays no attention to me if I give up trying my best. (T)

*Note.* *Participants were presented with father and (mother) versions of these items in Study 2. T: Task involving, E: Ego involving.
